# A Method for the *In Vivo* Measurement of Zebrafish Tissue Neutrophil Lifespan

**DOI:** 10.5402/2012/915868

**Published:** 2012-07-16

**Authors:** Giles Dixon, Philip M. Elks, Catherine A. Loynes, Moira K. B. Whyte, Stephen A. Renshaw

**Affiliations:** ^1^MRC Centre for Developmental and Biomedical Genetics, University of Sheffield School of Medicine, Sheffield S10 2RX, UK; ^2^Department of Infection and Immunity, University of Sheffield, Sheffield, UK

## Abstract

Neutrophil function is thought to be regulated, in large part, by limitation of lifespan by apoptosis. A number of studies suggest that circulating neutrophils have a half-life of approximately 6 hours, although contradictory evidence exists. Measuring tissue neutrophil lifespan, however, is more problematic. It is thought that tissue neutrophils survive longer, perhaps with a half-life in the order of 3–5 days, but this has never been directly measured. Zebrafish are an emerging model organism, with several advantages for the study of vertebrate immunity. In zebrafish, neutrophils constitutively assume tissue locations allowing their direct study *in vivo*. Using a transgenic approach, neutrophils were labelled with a photoconvertible pigment, Kaede. Photoconversion parameters were optimised and the stability of the Kaede confirmed. Individual neutrophils were photoconverted by scanning a confocal 405 nm laser specifically over each cell and their survival monitored for 48 hours, revealing an *in vivo* half-life for zebrafish tissue neutrophils of around 120 hours (117.7 hrs, 95% CI 95.67–157.8). Laser energy did not extend neutrophil lifespan, and we conclude that this represents a lower bound for the lifespan of a resting tissue neutrophil in the developing zebrafish larva. This is the first direct measurement of the lifespan of an *in vivo* tissue neutrophil.

## 1. Introduction

Neutrophils are key cells of the innate immune response, protecting against invading infectious agents. During inflammatory events, neutrophils are recruited to appropriate sites where they phagocytose and kill bacteria. In addition, they may release the contents of their intracellular granules into the local tissue environment. These granules contain reactive oxygen species and proteases that may contribute to tissue injury. Regulation of neutrophil function is therefore critical to ensure an appropriate balance between protection against infection and bystander host damage. Current understanding of inflammatory disease suggests that this balance can be perturbed in disease states. The predominant mechanism by which neutrophil function appears to be controlled is by regulation of lifespan. Circulating neutrophils are thought to either die by apoptosis in the circulation or to be targeted back to the bone marrow by upregulated CXCR4 expression where they are killed and recycled [1]. Determining the* in vivo *lifespan of neutrophils in the circulation and how it is regulated is therefore critical to understanding neutrophil production and homeostasis. In parallel, investigation of the regulation of *in vivo* lifespan of neutrophils in the tissues is essential for understanding how inflammation resolves. Modulation of the functional lifespan of the tissue neutrophil by endogenous and exogenous modulators of neutrophil apoptosis may underlie the perpetuation of inflammation and subsequent tissue damage seen in many inflammatory diseases; it may also be a key target for therapeutics that could limit tissue damage in inflammation while preserving host defense functions of the neutrophil. These two questions therefore are currently receiving much attention in the literature.

Measures of the lifespan of circulating human neutrophils labeled via a variety of techniques give estimates of neutrophil half-life of 6 hours (summarized recently in [2]). However, recent advances using human neutrophils labeled *in vivo* report the circulating lifespan to be approximately 5 days [3], but this has recently been questioned by a number of authors [2, 4].

In contrast, it has proved much more difficult to address the arguably more important question of the lifespan of tissue neutrophils. It has not, to date, proved possible to measure this directly, but *in vivo *labeling studies, for example, using 18FDG [5, 6], suggesting tissue neutrophil lifespan is prolonged compared to circulating neutrophils.

The uncertainty as to the *in vivo* fates of individual cells relates in part to the difficulty in following individual immune cells over the course of their lifespan *in vivo*. Zebrafish are emerging as a powerful model for the understanding of vertebrate immunity, and several important advances have recently been made that demonstrate the unique advantages of the zebrafish system for the study of immunity (recently reviewed in [7]). Transgenic zebrafish models allow direct visualisation and tracking of individual cells, and of populations of cells, allowing their fate to be determined *in vivo*. Unlike mammalian systems, zebrafish neutrophils adopt a tissue location constitutively and migrate to sites of inflammation, allowing direct visualisation of the lifespan of tissue neutrophils *in vivo*. Using a novel *in vivo *zebrafish model, in which the fluorescent protein Kaede, notable for its ability to change colour on exposure to light, is expressed in neutrophils, the lifespan of tissue neutrophils *in vivo* has been measured. We find a lower bound for tissue neutrophil half-life of approximately 95 hours, in keeping with existing indirect measures.

## 2. Methods

### 2.1. Reagents, Zebrafish Lines, and Maintenance

All reagents were from Sigma-Aldrich (Poole, UK) unless otherwise stated. Zebrafish were maintained according to standard protocols [8]. The *Tg(lyz:Gal4)i252* [9], *Tg(Actin:Gal4)* [10] and *Tg(UAS:Kaede)s1999t* [11] lines are described elsewhere.

### 2.2. Microscopy, Photoconversion, and Image Processing

For confocal microscopy, a Perkin Elmer Ultra*VIEW* VoX ERS 6FR Laser Confocal Imaging System (Perkin Elmer INC, USA) with an inverted Olympus IX81 microscope, equipped with six diode laser lines and a Yokogawa CSU-X1 spinning disk was used to capture images on a 14-bit Hamamatsu C9100-50 Electron Multiplying-Charged Couple Device (EM-CCD) peltier-cooled camera (Hamamatsu Photonics Inc.), through an appropriate filter. A Perkin Elmer Ultra*VIEW* PhotoKinesis device, attached to the microscope described above, was used to photoconvert the Kaede protein using a 405 nm laser line. All photoconversion of samples was performed using an Olympus 10X objective lens (UPlanSApo NA 0.4). The device was calibrated using a glass microscope slide (Menzel-Gläzer) covered with fluorescent highlighter ink (Stabilo Boss) as a photo-bleachable substrate (according to manufacturer's instructions). Photoconversion was performed on agarose-mounted larvae using 40% laser power for 120 cycles of the 405 nm laser line. Z-stacks were taken immediately pre- and post-photoconversion. The larvae were then released from the agarose and transferred to fresh embryo media. The petri dishes containing the larvae were wrapped in aluminum foil to prevent background photoconversion. At the timepoints indicated, larvae were again mounted in low-melting point agarose and Z-stacks taken using a 2x objective lens, and neutrophils counted. For fluorescence microscopy, a Nikon Eclipse TE2000-U Inverted Compound Fluorescence Microscope (Nikon UK Ltd) was used with a Hamamatsu 1394 ORCA-ERA (Hamamatsu Photonics Inc.). Images were captured using Volocity build 5.3.2 through a 2x Nikon Plan UW NA 0.06 objective. Multiple Z-stacks were acquired using a Prior Nanoscan-Z 200 *μ*m piezo *Z*-axis drive.

### 2.3. Statistical Analysis

Unless otherwise stated all graphs were generated and statistical analysis performed using GraphPad Prism 5 software. Significance was assumed for *P* < 0.05.

## 3. Results and Discussion

### 3.1. Optimisation of Photoconversion Parameters

**Figure 1:**
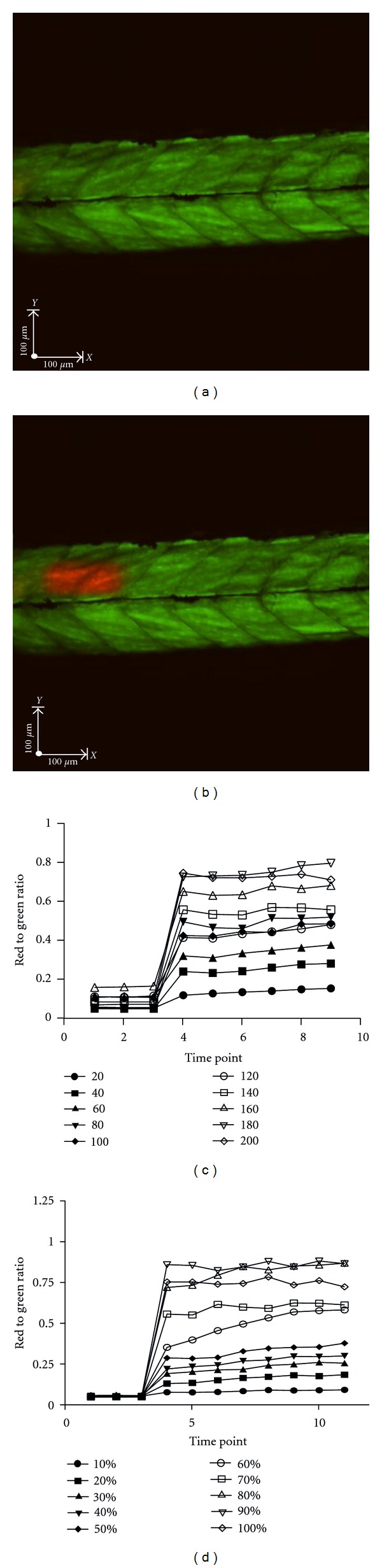
Optimisation of Kaede photoconversion parameters in muscle. (a) Photomicrograph of a 3 dpf zebrafish compound transgenic larva (Actin:gal4 × UAS:Kaede) prior to photoconversion showing green Kaede protein, and no red fluorescence. (b) The same larva as (a) after photoconversion, showing rectangular red area of photoconverted Kaede, photoconversion was achieved using 40 cycles of 40% 405 nm laser power. (c) and (d) The effect of varying the number of cycles (c) and laser power. (d) of the 405 nm Photoconversion laser on the change in ratio of red to green fluorescence during photoconversion. Gal4 UAS:Kaede larva, similar to that shown in (a). When not varied, 120 cycles or 40. Photoconversion was carried out on a small area of tissue in an actin: % laser power were used.

To accurately monitor the behaviour of individual cells *in vivo* over time requires the ability to label chosen cells with a stable tag that can consistently be identified over time and that does not influence the parameter being measured. The photoconvertable protein, Kaede, is potentially suitable for this use since it can be readily converted from green to red fluorescence by light exposure. This can be achieved at the level of single cells using the laser scanning capacity of confocal microscopes. We aimed to use neutrophil-specific expression of Kaede to photoconvert individual neutrophils *in vivo* and to follow their fate over several days. The minimum amount of laser energy required was identified using a series of optimisation experiments in muscle cells, which do not migrate and, unlike neutrophils, are thought not to be regulated by apoptosis. In fish from a cross of Actin:Gal4 and UAS:Kaede, regions of muscle were photoconverted using the PhotoKinesis device on an ultraview spinning disk confocal microscope as detailed in Section 2 (Figures 1(a) and 1(b)). Two parameters were tested concerning the photoconversion: number of cycles of photoconversion and power of the 405 nm photoconversion laser.

Timelapse video microscopy was performed to capture 8–12 timepoints. The intensity of red and green fluorescence at each point was measured and tracked over time. The ratio of red to green fluorescence was used to analyse the degree of photoconversion. The degree of photoconversion was increased by a change in the number of cycles of photoconversion up to 180 cycles, after which the increase was not maintained (Figure 1(c)). An increase in the laser power used for photoconversion also gives an increase in the ratio of red to green fluorescence. The increase in ratio is preserved over the range of photoconversion laser power up to 80%, after which there is a fall off of photoconversion efficiency (Figure 1(d)). Using these data, and other optimisation experiments (not shown), compromise photoconversion parameters were chosen which most reliably balanced the ability to clearly observe converted cells, with the minimal laser energy delivered, and the shortest duration of photoconversion. More cycles of conversion at lower energies could lead to prolonged experimental protocols, which might have a detrimental influence on the outcome of the experiment. For subsequent experiments 120 cycles of 40% 405 nm laser power were used.

Levels of photoconversion efficiency were well below the published values of 2000 fold increase in the red to green fluorescence ratio [12]. In the published literature, purified Kaede protein is photoconverted *in vitro *allowing for uniform photoconversion. This was not possible in a live *in vivo* model with Kaede expressed in the musculature. Using scanning confocal lasers delivers the photoconversion energy concentrated at the centre of an “egg timer” shape in Z, thus assessments of whole-cell fluorescence will include sub-optimally converted tissue, thereby reducing the perceived efficiency of photoconversion. In addition, photoconversion is highly sensitive to irradiation with UV or violet light between 350 and 400 nm. The only wavelength of laser available for photoconversion in our laboratory was the 405 nm laser line. It is possible that this laser wavelength does not provide optimal photoconversion.

### 3.2. Photoconverted Kaede Is Stable for at Least 48 Hours

It is clear Kaede could potentially provide a powerful tool for tracking individual cells *in vivo*. However, if Kaede is to be used to follow individual cells over long periods of time, it is important that photoconverted Kaede remains stable for the timespan of the experiments. It has been reported previously that photoconverted Kaede remains stable for months; however, this is in a purified state and not *in vivo* [12]. Photoconverted Kaede needs to remain distinguishable from nonphotoconverted Kaede for at least 48 hours, the maximum timespan of these experiments.

**Figure 2:**
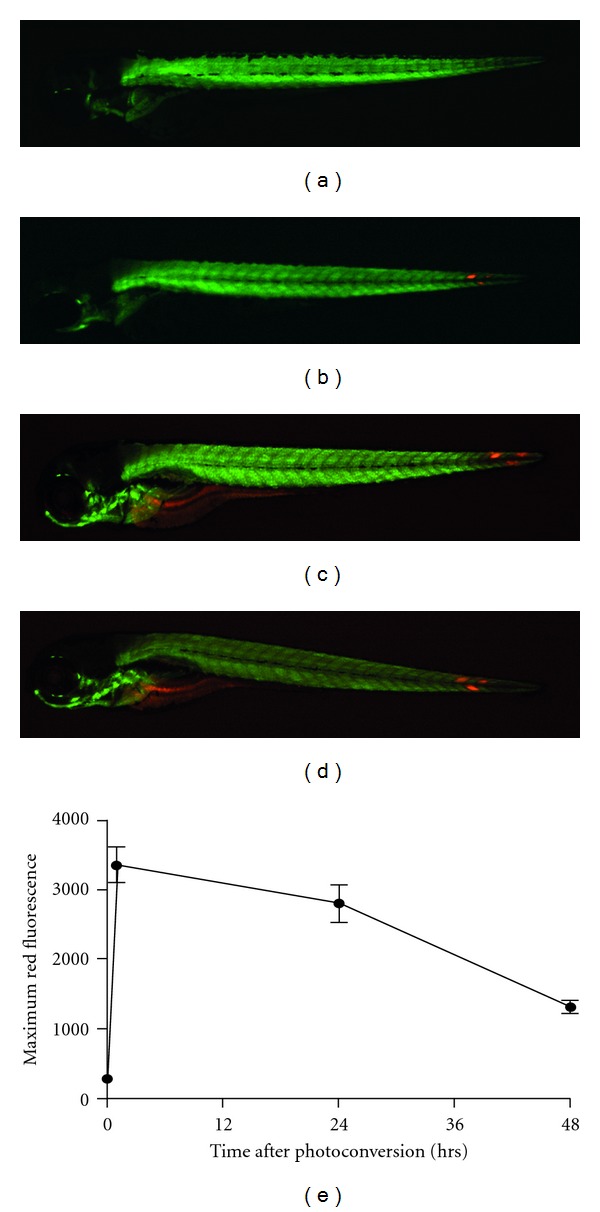
CCPhotoconverted Kaede remains visible for at least 48 hrs postphotoconversion. (a)–(d) Images are representative examples of 8 fish, all fish showed the same pattern. Individual muscle blocks were photoconverted using 120 cycles of 40% 405 nm laser power. (a) 3 dpf Actin:Gal4 × UAS:Kaede larva prior to photoconversion shows no red fluorescence. (b) Larva 1 hour after photoconversion showing 3 distinct areas of photoconversion. (c) 24 hours after photoconversion. (d) 48 hours after photoconversion. For these images exposures were chosen to optimise the image quality, and are not comparable to those used for optimisation. (e) Analysis of the intensity of fluorescence of the red photoconverted Kaede over 48 hours. Kaede protein within the Actin:Gal4 × UAS:Kaede larvae was photoconverted with 120 cycles of 40% 405 nm laser power. Maximum red fluorescence was defined as the highest intensity of red fluorescence found in the photoconverted portion of the larva. *n* = 8.

In order to assess the stability of photoconverted Kaede, 3 distinct sections of tissue were photoconverted in actin:Gal4 × UAS:Kaede larvae. The mounted larvae were then released from the agarose and then remounted at 24 and 48 hours after photoconversion. The photoconverted tissue was imaged at each timepoint using identical acquisition settings, so as not to create any bias. We observed that photoconverted cells persist over the experiment period of 48 hours (Figures 2(a)–2(d)). The intensity of red fluorescence (from the photoconverted Kaede) was then measured over this time period. The maximum intensity of red fluorescence decreases over the 4 hour timespan following photoconversion (Figure 2(e)). Larvae were imaged prior to photoconversion at “timepoint 0.” Following the initial increase in fluorescence (photoconversion), the intensity was reduced over the subsequent 48 hours. However, the maximum red fluorescence at 48 hours was still significantly higher than before photoconversion. Importantly, the photoconverted tissue was easily distinguishable from non-photoconverted tissue at every point over the 48 hour period.

### 3.3. Measuring the Lifespan of Zebrafish Tissue Neutrophils

**Figure 3:**
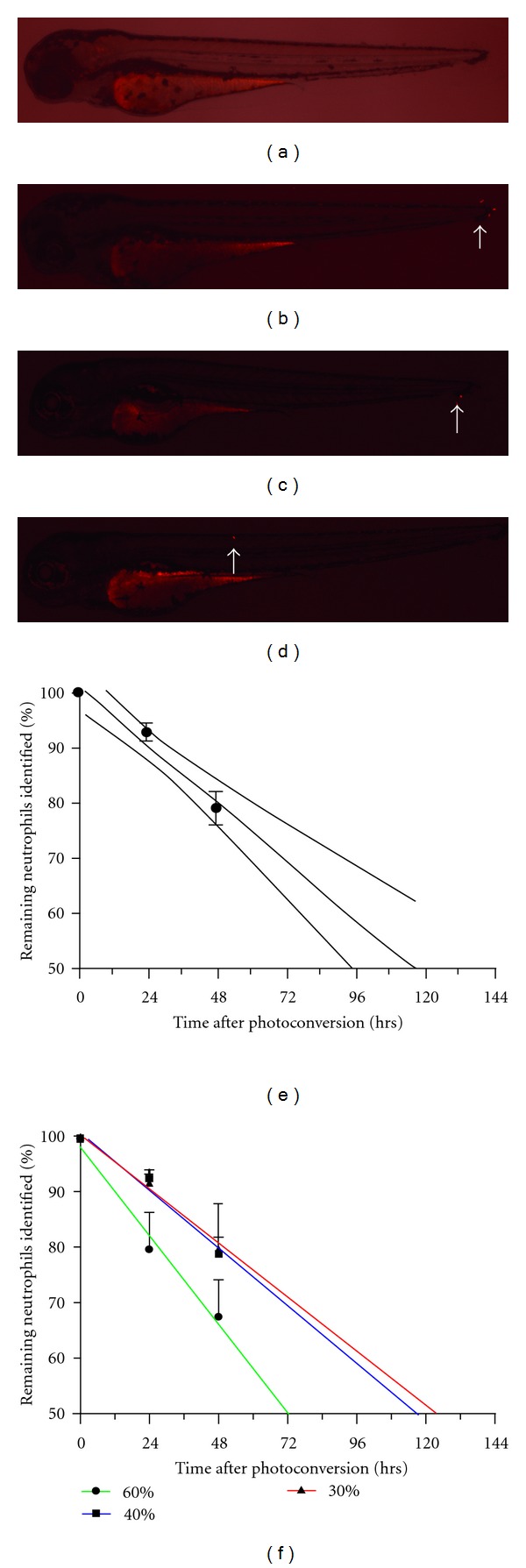
Direct *in vivo* measurement of tissue neutrophil lifespan. Neutrophils were photoconverted using the PhotoKinesis device on the Laser Confocal Imaging System (Perkin Elmer Inc.). 40% 405 nm laser power for 120 cycles was used to photoconvert 3 individual tissue neutrophils in each of 32 fish at 3 dpf. The images shown above were selected as examples of larvae with a range of photoconverted neutrophils visible. Images were selected from an image series covering 0–48 hours after photoconversion. Images are Z-stacks taken using a 2x objective. (a) Larvae with no photoconverted neutrophils visible. (b) Larva with 3 photoconverted neutrophils present in the tail region. (c) Larva with 2 photoconverted neutrophils present in the tail region. (d) Larva with one photoconverted neutrophil on the dorsal aspect. (e) The number of photoconverted neutrophils was counted from Z-stack images taken at 0, 24, and 48 hours after photoconversion using a 2x objective lens. 160 larvae were used in total over 5 experimental repeats. The data were fitted with a linear regression, with error bars indicating standard error mean (SEM). (f) The number of photoconverted neutrophils was counted from Z-stack images taken at 0, 24, and 48 hours after photoconversion using a 2x objective lens. 30% and 60% laser power was repeated twice, and the 40% laser 5 times resulting in a total of 288 larvae being included in the analysis. *P* < 0.05 for the difference between remaining neutrophils identified at 24 hours for 60% laser power and 40% and 30% laser power (2-way ANOVA). There is no significant difference in the percentage neutrophils remaining at 48 hours after photoconversion. Mean ± SEM is shown.

The lifespan of a tissue neutrophil in any species has never previously been directly measured. In order to calculate a half-life for tissue neutrophils, photoconversion of limited numbers of tissue neutrophils in unchallenged fish from a cross of lyz:Gal4 and UAS:Kaede was performed using the PhotoKinesis device, and the persistence of these cells measured. Neutrophils chosen for study were identified away from the posterior blood island to avoid labeling of dividing neutrophil precursor cells [13]. An increase in photoconverted neutrophil number over the timecourse of the experiment was not observed in any of the larvae. This is suggestive that division of neutrophils was not a major factor during these experiments, although we cannot exclude a small contribution from this phenomenon. Care was taken to ensure single cells only were photoconverted; this is particularly important as any overlapping cells would lead to an overestimate of lifespan, and this precludes reliable measurement of the lifespan of neutrophils involved in the inflammatory process where neutrophil density is significantly higher. The fish were imaged at 24 and 48 hours after photoconversion and the number of photoconverted neutrophils remaining were counted (Figure 3(e)). Using data acquired from 160 larvae, the half-life of a zebrafish tissue neutrophil during the 48 hour measurement window was found to be in the region of 120 hours (117.7 hrs 95% confidence interval 95.67–157.8). This is in keeping with tissue neutrophil lifespans inferred from mammalian data. In a rabbit model of lobar streptococcal pneumonia, and in human subjects with pneumonia, neutrophil influx ceased within the first day but metabolically active neutrophils continued to be detected for several days [5, 6]. We were unable to find any direct measures of tissue neutrophil lifespan *in vivo*. Since the measured half-life is longer than the duration of the measurement, this cannot be used to infer the behaviour of neutrophils beyond the end of the period of measurement. Moreover, this figure does not take into consideration the age of the neutrophil at photoconversion.

The thermal energy delivered by the photoconversion process itself could potentially alter the lifespan of the targeted neutrophils. These experiments were therefore repeated using variable laser energies to photoconvert the neutrophils. Increased photoconversion energy (60% laser power) led to shorter measured lifespans, but reduced photoconversion energy (30% laser power) did not lead to extended survival (Figure 3(f)). Photoconversion energies below 30% laser power were inadequate for visualisation of photoconverted neutrophils. These data suggest that, at these levels of photoactivation energy, the experimental procedure itself has a minimal impact on neutrophil lifespan and that the effect of laser power would be to shorten neutrophil lifespan rather than extend it. For this latter reason, we can be confident therefore that the lifespan of a resting neutrophil is likely to be at least that measured here.

While it would be highly interesting to define the lifespan of a neutrophil involved in the inflammatory process, this did not prove possible using this model, as we were unable to remove the possibility of photoconverting multiple cells unintentionally.

To our knowledge, the lifespan of vertebrate tissue neutrophils has not been measured before. However, a number of measures of mammalian circulating neutrophil lifespan have been made, by labeling* ex vivo *or* in vivo*. All measures until recently have given consistent results, with a half-life of 6–8 hours [2]. This is also in keeping with the clinical observation of the onset of neutropenia following bone marrow ablation. However, recent studies using labelled water and complex mathematical analysis have suggested that human neutrophil lifespans might be as long as 5 days [3]. Several authors have argued that these experiments have been misinterpreted, and advances in technology will be required before the true *in vivo* circulating neutrophil lifespan can be determined without doubt [2, 4].

## 4. Conclusion

The resting tissue neutrophil in the zebrafish is a relatively long-lived cell (compared to circulating mammalian neutrophils) having a half-life of approximately 5 days. This is, as far as we are aware, the first direct measure of tissue neutrophil lifespan *in vivo*, and is in agreement with indirect and inferred measures from mammalian systems.
